# Identification of circulating extracellular vesicle long RNAs as diagnostic biomarkers for patients with severe acute pancreatitis

**DOI:** 10.1002/ctm2.1034

**Published:** 2022-10-21

**Authors:** Qingfu Zhu, Rui Yang, Jiaxin Luo, Hao Xu, Hengrui Li, Xiaoling Liu, Ke‐Qing Shi, Hui‐Ping Li, Fei Liu

**Affiliations:** ^1^ Eye Hospital, School of Ophthalmology & Optometry, School of Biomedical Engineering Wenzhou Medical University Wenzhou Zhejiang China; ^2^ The First Affiliated Hospital of Wenzhou Medical University Wenzhou Zhejiang China

Dear Editor,

Severe acute pancreatitis (SAP) may cause multiple organ dysfunction and results in the systemic inflammatory response syndrome.^1^ The contrast‐enhanced computed tomography (CECT) is the main diagnostic method in clinical practice, but has several disadvantages such as time delay, missed diagnosis and high false‐positive rate, and renal burden caused by contrast agents.^2^ Since SAP has a high mortality, the novel detection methods and non‐invasive biomarkers are urgently needed for timely, rapid, and precision SAP diagnosis.

Small extracellular vesicles (sEVs), transport of biological molecules (RNAs, proteins, and metabolites) from disease‐related organs or tissues to target cells for cell‐to‐cell communication has been demonstrated as a potential source for biomarker discovery.^3^ Here, we have performed a transcriptome analysis of sEVs circulating in blood and integrated transcriptomics and metabolomics to identify the commonly enriched pathways for discovering potential biosignatures associated with acute pancreatitis (AP) severity (Figure 1). The AP patients were diagnosed using computed tomography (CT) (Figure S1) and the isolated sEVs using EXODUS^4^ were characterised with regard to their particle size, morphology, and protein markers according to MISEV2018 guidelines,^5^ which shows good quality of vesicle product for downstream analysis (Figure S2).

**FIGURE 1:**
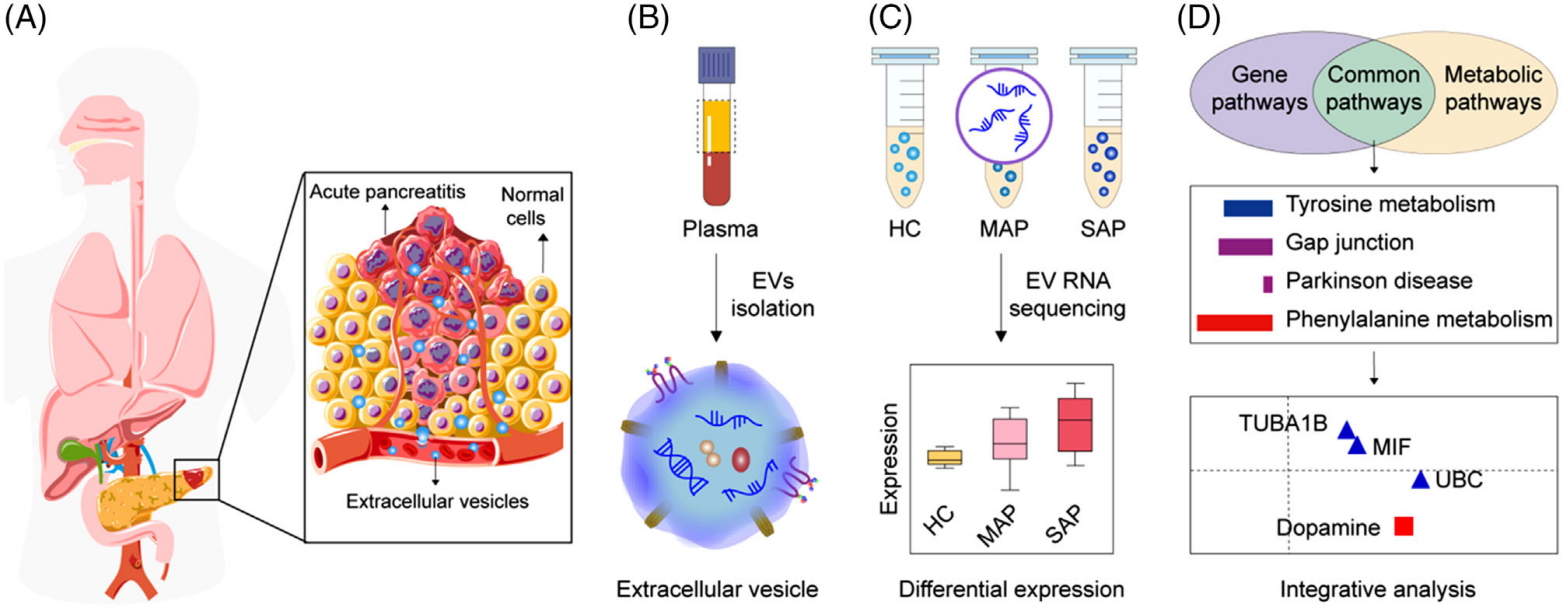
Transcriptomic analysis of circulating extracellular vesicles (EVs) for severe acute pancreatitis (SAP) diagnosis. (A) EVs are secreted by pancreatic tissue entering the blood circulation. (B) EV isolation using EXODUS. (C) EV transcriptomic analysis using RNA sequencing. (D) Identification of the key regulators from MAP to SAP by integrative analysis of EV transcriptomics and metabolomics.

We then depicted the transcriptomic profiles of circulating sEVs by long RNA sequencing from groups of SAP, mild acute pancreatitis (MAP) and healthy controls (HC). A total of 58,830 genes were detected, from which there were 34,245 mRNAs and 7,665 long noncoding RNAs (lncRNAs), accounting for 58.2% and 13.1%, respectively (Figure 2A). The three groups, MAP, SAP, and HC display significantly distinct long RNA profiles and show 647, 1,258, and 1,138 differentially expressed genes (DEGs) (log2(FC) > 4 and *p* < 0.01) for the following comparisons: MAP *vs*. HC, SAP *vs*. HC and MAP *vs*. SAP, respectively (Figures 2B,C, S3, 4, and Tables S2, 3), and their enriched pathways are very likely to be associated with the development of AP showing in Figure 2D,E. We also investigated the co‐expression relationship between differential lncRNA and mRNA and conducted enrichment analysis including Kyoto Encyclopedia of Genes and Genomes (KEGG) analysis (Figure 2D,E), mRNA interaction network analysis (Figures S3B,C and S4B,C), Gene Ontology (GO) clustering analysis (Figures S3D and S4D). The results are highly related to AP pathogenesis evidenced by the enriched pathways such as gap junction, aldosterone synthesis and secretion pathways with the target genes of lncRNA for MAP group, and HIF‐1 signalling pathway, natural killer cell‐mediated cytotoxicity, fat digestion and absorption for SAP group (Figure 2D,E).^6^, ^7^

**FIGURE 2:**
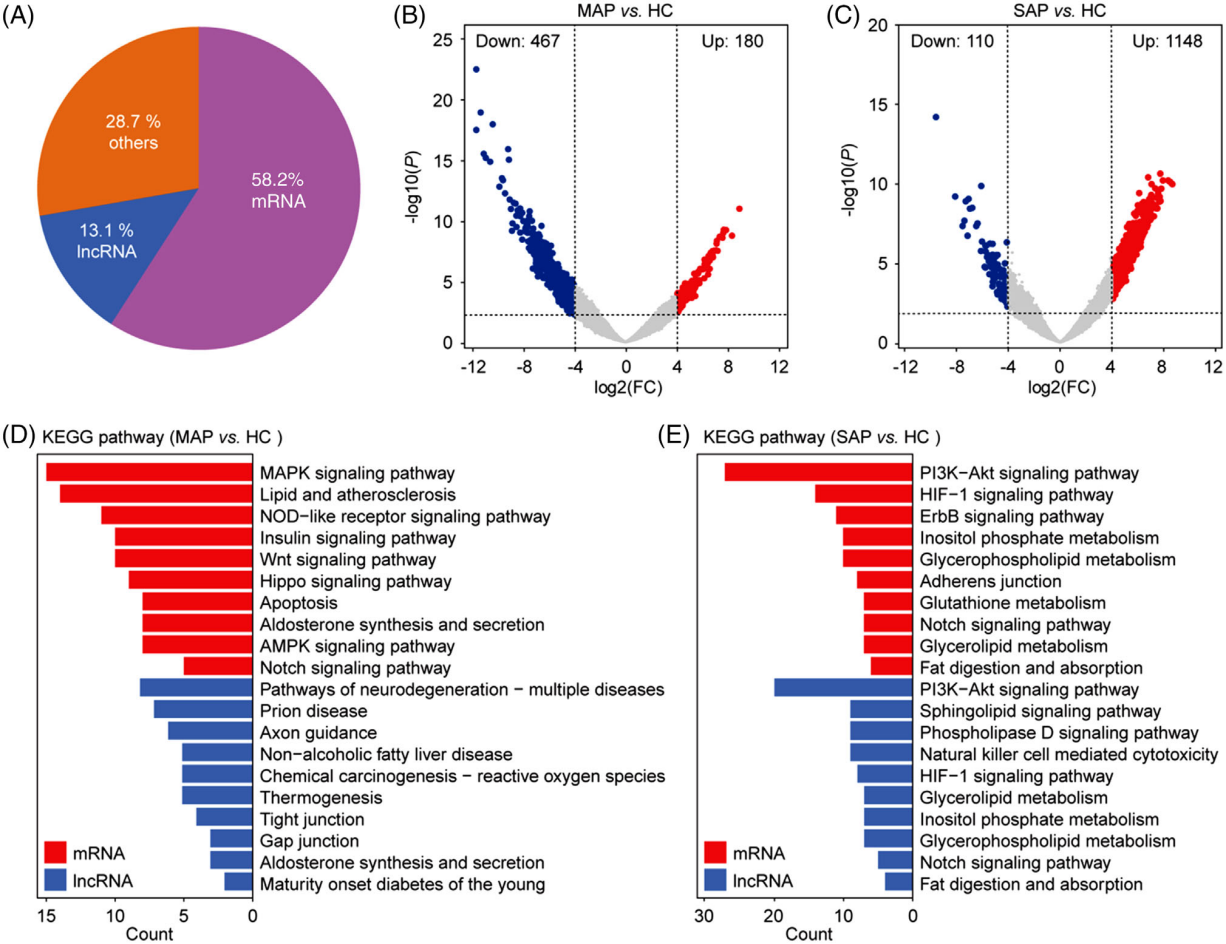
Comparative analysis of long RNA profiles of acute pancreatitis and healthy controls. (A) Distributions of RNA biotypes identified from RNA sequencing, including long noncoding RNA (lncRNA), protein coding RNA (mRNA) and others. (B) The volcano diagram shows up‐ and down‐regulated genes in MAP and (C) SAP compared to HC (*p* < 0.01; log2(FC) > 4). (D) Enriched KEGG pathway in MAP group and (E) SAP group based on the differentially expressed genes. Red: mRNA gene enrichment pathway; blue: lncRNA target gene enrichment pathway.

We further analyzed the potential of circulating sEVs to distinguish SAP from MAP. We observed 1,119 up‐regulated genes (106 lncRNAs and 1,013 mRNAs) and 19 down‐regulated genes (2 lncRNAs and 17 mRNAs) in SAP group compared to MAP group (Figure 3A ,Figure S5A and Table S4). The top 20 DEGs of two groups are shown in the heatmap in Figure 3B and Table S4, and the interactions between differential lncRNAs and mRNAs are presented in Figure S5B. It can be seen that the overall expression level of DEGs in SAP group was much higher than that in MAP group. Figure 3C shows the high‐confidence network (>0.9) of interaction relationships among these target genes. UBC displayed the highest degree of interaction with other genes. We then analyzed the function of DEGs through KEGG analysis (Figure 3D,E) and GO analysis (Figure S5C) and found that the enrichment pathways based on long RNAs included C‐type lectin receptor signalling pathway, T‐cell receptor signalling pathway,and B‐cell receptor signalling pathway. These pathways are mainly related to immune responses, that is, during the process of MAP to SAP, immune cells are activated and release more inflammatory factors, leading to more severe inflammatory responses.

**FIGURE 3:**
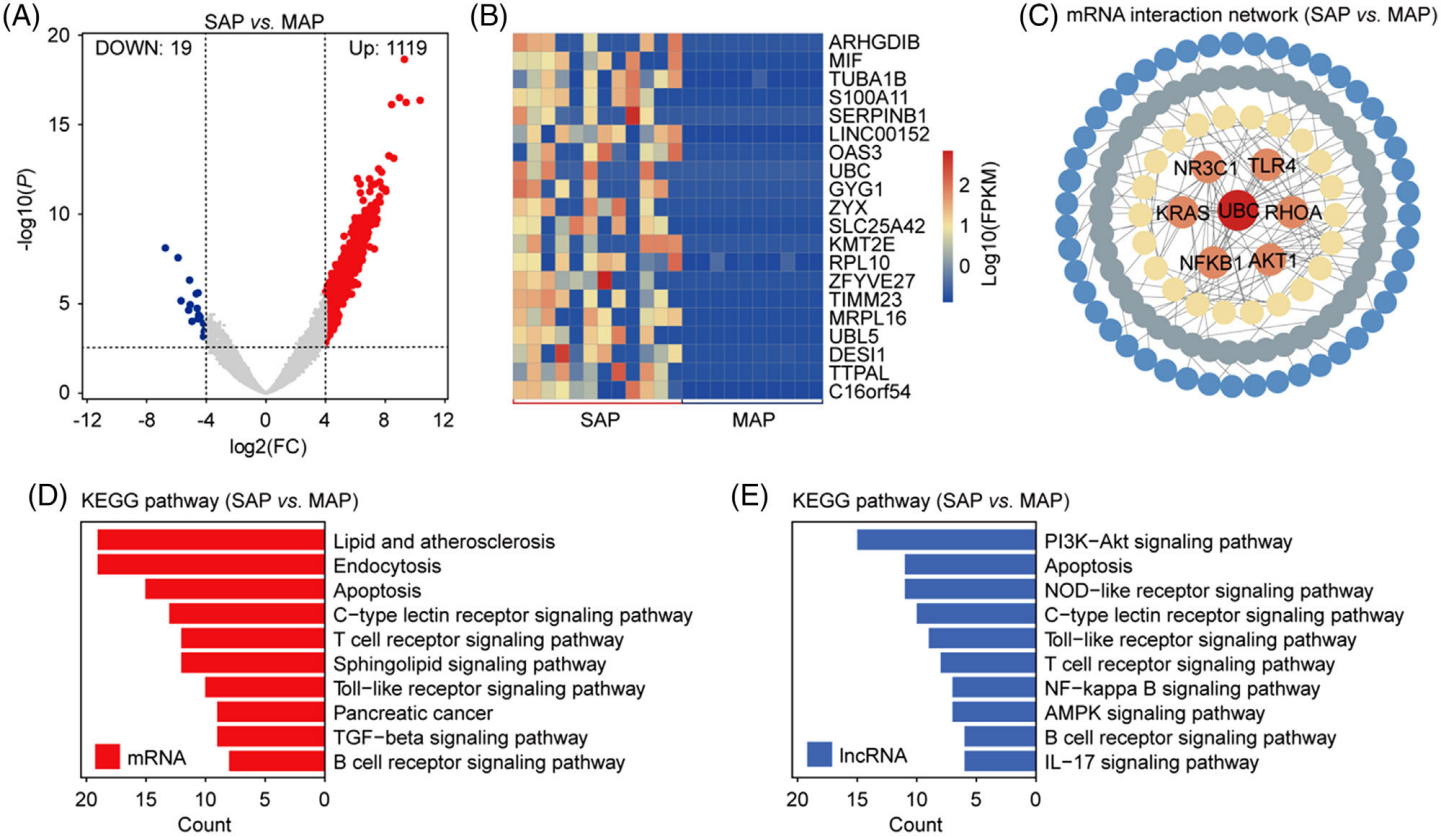
Comparative analysis of RNA expressions in severe acute pancreatitis (SAP) and mild acute pancreatitis (MAP). (A) The volcano diagram shows the up‐ and down‐regulated genes in SAP compared to MAP. (B) The hierarchical cluster diagram shows the top 20 differential genes sorted by *p*‐value ascending. (C) mRNA interaction network between SAP and MAP. (D) Enriched KEGG pathways based on differential mRNA of SAP and MAP. (E) The enriched KEGG pathways based on lncRNA target genes of SAP and MAP. Red: mRNA gene enrichment pathway; blue: lncRNA target gene enrichment pathway.

To obtain precision biosignatures for assessing the severity of AP, we further integrated the transcriptional and corresponding metabolic profiles to investigate their common pathways. This will allow us to select the differential genes that mostly interact with metabolites by tracking the downstream changes in metabolic products to discover the key regulatory genes in the AP development. The top 20 differential mRNAs and top 20 differential lncRNAs were selected as the main research objects (Table S4), and the differential metabolites was recognised based on our previous work (Table S5).^8^ Figure 4A visually shows the correlation heatmap between differential metabolites and differential genes obtained by the Spearman algorithm, showing the strong positive connections between transcriptomics and metabolomics. Via co‐enrichment analysis, we subsequently obtained the significant interconnections between genes and metabolites (Figure 4B), pointing to the common pathways of tyrosine metabolism, gap junction, Parkinson's disease, and phenylalanine metabolism.

**FIGURE 4:**
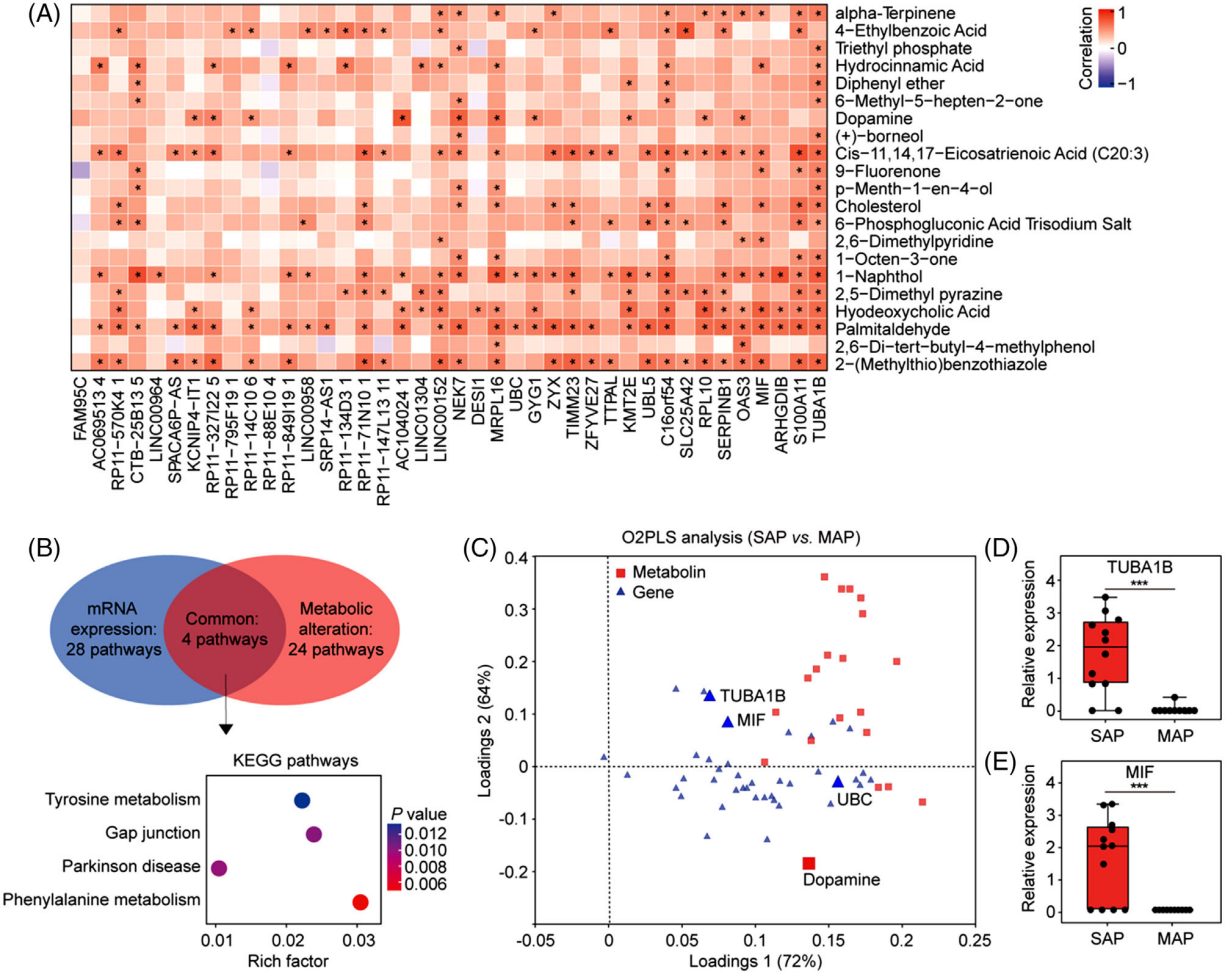
The integrated analysis of sEV transcriptomics and metabolomics for SAP diagnosis. (A) The heatmap visually shows a significant positive correlation between differential genes and differential metabolites between SAP and MAP. (SAP, n = 12; MAP, n = 10; *p* < 0.05). (B) KEGG enrichment analysis revealed four common pathways between genes and metabolites. (C) O2PLS analysis shows a trend of deterministic model differences between genes and metabolites. (D) Relative expression levels of TUBA1B (*p* = 0.0002) and (E) MIF (*p* = 0.0009).

We further looked into the expression levels of genes and metabolites involved in these common pathways via O2PLS analysis, and found that MIF, TUBA1B, UBC and dopamine were significantly enriched and upregulated in tyrosine metabolism, gap junction, and phenylalanine metabolism in SAP compared with MAP (Figure 4C). TUBA1B and dopamine coexist in the gap junction pathway and previous studies have shown that the gap junction can promote the exchange of ions and small molecules between cells,^9^ suggesting that they have important functional roles in the regulation of sEVs transport. Notably, TUBA1B, UBC, and dopamine were upregulated simultaneously, although they showed low correlations. MIF is a class of chemokines which has been previously reported for prediction of pancreatic necrosis in AP,^10^ and our data show that the sEV MIF was involved in the metabolism of tyrosine and phenylalanine, and was significantly positively correlated with benzene, lipid, and organic acid metabolites such as 2‐(methyl thio‐benzothiazole), palmitaldehyde, hyodeoxycholic acid and 1‐naphthol. Figure 4D,E shows the significant differences of TUBA1B and MIF with regard to their expression levels between SAP and MAP, respectively, indicating an excellent discriminative power of these two genes.

In summary, we have investigated the alternations of transcriptome profile of plasma‐derived sEVs from AP patients. The integrated analysis of transcriptomics and metabolomics provide a comprehensive view of cell conditions, which indicates the MIF and TUBA1B may be the key variables that play an important role in the AP progressions. Thus, these two DEGs may serve as potential markers for SAP diagnosis. We believe that the regulatory relationship between genotype and phenotype is of great significance in the study of SAP mechanism towards clinical translations.

## CONFLICT OF INTEREST

The authors declare they have no conflicts of interest.

## Supporting information



Supplementary information
