# Supplementary material for: Identification of circulating extracellular vesicle long RNAs as diagnostic biomarkers for patients with severe acute pancreatitis
Source: Clin Transl Med. 2022 Oct 21;12(10):e1034. doi: 10.1002/ctm2.1034 (PMC9587378; doi:10.1002/ctm2.1034)
Supplement: Supplementary file 1 — Supplementary information [file CTM2-12-e1034-s001.docx]

**Identification of circulating extracellular vesicle long RNAs as diagnostic biomarkers for patients with severe acute pancreatitis**

Qingfu Zhu^1#^, Rui Yang^1#^, Jiaxin Luo^1^, Hao Xu^1^, Hengrui Li^1^, Xiaoling Liu^1^, Keqing Shi^2^ Hui-Ping Li^2^, Fei Liu^2*^

^1^Eye Hospital, School of Ophthalmology & Optometry, School of Biomedical Engineering, Wenzhou Medical University, Wenzhou, Zhejiang 325035, China

^2^The First Affiliated Hospital of Wenzhou Medical University, Wenzhou, Zhejiang 325000, China

^#^Equal contribution authors

*Corresponding author: feiliu@wmu.edu.cn (Fei Liu)

**Methods**

**Clinical samples**

All subjects were from the First Affiliated Hospital of Wenzhou Medical University (12 SAP patients, 10 MAP patients, and 8 healthy people) following a protocol approved by the Institutional Review Board of the First Affiliated Hospital of Wenzhou Medical University (Wenzhou, China) and gave their informed consent. All patients were diagnosed according to the revised Atlanta classification and definition. Blood samples were collected from patients immediately after admission, and plasma samples were centrifugally collected (15min, 1,500g) and stored at −80 ^o^C until use.

**EV sample preparation**

EVs were isolated and purified from 200 μL plasma samples using the EXODUS device. First, the plasma samples were diluted 75 times with phosphate-buffered saline (PBS) to a final volume of 15 mL. Then, a 0.22 μm membrane filter (Millipore) was used for filtration. An EXODUS device with a 25 mm diameter AAO membrane (Whatman Anodisc inorganic filter membrane, pore size 20 nm) was used to collect EV particles, which were then resuspended in 200 μL PBS and stored at −80 ^o^C until use.

**Nanoparticle tracking analysis (NTA)**

NTA was performed according to the manufacturer's instructions. The EV size distributions and vesicle concentrations were characterized by a NanoSight NS300 (Malvern) equipped with a 488 nm laser and a highly sensitive sCMOS camera. The samples were diluted appropriately and introduced into the instrument with micropumps. The particles per frame value were 30~50. Each sample was measured 3 times and the capture time was 30 seconds.

**Western Blot (WB) analysis**

The purified sEVs were quantified using a Nanodrop 2000 (Malvern panalytical, China). The EV proteins were separated by electrophoresis using 4-20% prefabricated polyacrylamide plate microgel (Tris-glycine, pH 8.3) via sodium dodecyl sulfate-polyacrylamide gel (SDS-PAGE) and transferred to polyvinylidene fluoride (PVDF) membrane. The PBST (0.5%Tween-20) with 5% non-fat dry milk was used as a blocking medium at room temperature for 1 h and the PVDF membranes were then incubated with primary antibodies at 4 ^o^C overnight. The following antibodies were diluted at 1:1000: Alix (Santa Cruz), anti-Flotillin1 (BD), and anti-CD9(Santa Cruz). The membrane was washed 5 times with PBST (0.1%Tween-20) at pH 7.4 for 6 min and then incubated for 60 min with enzyme-conjugated anti-mouse IgG or enzyme-conjugated anti-rabbit IgG as the secondary antibody (1:3000) at room temperature. The protein band was visualized by the Enhanced Visualization of Chemiluminescence immunoassay imaging (Pei Qing, China)

**Transmission electron microscope (TEM) analysis**

20 μL of purified EV samples were mixed with 4% paraformaldehyde (PFA) (1:1, v/v), which was then transferred to the carbon mesh and incubated for 30min. The carbon mesh adsorbed with sEV particles was then washed with 100 μL of PBS for 2min (6 times). Next, the vesicles were fixed with 50 μL of 1% glutaraldehyde for 5 min and then washed with 50 μL of ultra-pure water (Milli-Q) (repeat 6 times, 2 min each). After that, the sEV particles were negatively stained with uranyl acetate (2%) for 30 seconds. The samples were dried and observed by transmission electron microscope (Talos F200S, Thermo).

**RNA-sequencing analysis**

Total sEV RNA was isolated using the RNeasy Mini Kit (Qiagen) according to the manufacturer's instructions. In brief, 140 μL of chloroform was added to the EV sample, and the mixture was centrifuged at 12,000g for 15min. The upper water phase was added with 1.5 times the volume of anhydrous ethanol and transferred to the RNeasy adsorption column for RNA purification. We prepared the RNA-seq Library using SMARTer® Starfish Total RNA-seq Kit Pico Input Mammalian (Clontech). According to the instructions of the RNALib single-cell WTA kit, 4 µL samples were taken for microamplification with 18 amplification cycles, and cDNA amplification products were purified by magnetic beads. According to the Lifeint Transpose DNA Library Prep Kit for Illumina instruction, 1 ng cDNA was taken for Library construction, and the number of amplification cycles was 14. The final RNA Library was obtained after purification by magnetic beads. An average of 150bp paired terminal sequences were obtained from 30 samples by RNA-seq.

The Trim_galore software was used to delete row data reads and Hisat2 was used to map high-quality reads with the Human Reference Genome (GRCH38). The gene expression profile of FPKM was calculated by Stringtie software. The differential genes were identified by comparing Bioconductor Package (DESeq2 Version 1.32.0) and R (Version 4.1.0) software, and the fold change and *p* value were calculated according to the count value. Gene expression level at zero in half of the samples in a group were not considered differential expressions. The enrichment analysis was completed by Cluster Profiler (Version 4.2.2R) and the direct mapping was performed using the R Studio mapping package (Version ggploT2 3.3.5). The Spearman correlation test was used to calculate the co-expression relationship between lncRNA and mRNA. The PPI network was obtained by importing potential genes into the String database (https://cn.string-db.org/).

**Integrative analysis of transcriptomics and metabolomics**

All metabolome data were derived from our previous work^15^. The ggplot2 package (version 3.3.5R), Venn diagram (Version 1.6.20R), and SIMCA software (Version 16.0.2, Sartorius Stedim Data Analytics AB, Umea, Sweden) are used for graphic rendering. The screening thresholds of differential metabolites were as follows: the absolute value of FC > 1.2 and *p* < 0.05. Spearman correlation analysis was used to calculate the co-expression relationship between top 20 genes and differential metabolites. KEGG comments were extracted from the official website. The O2PLS was automatically modeled and the data was analyzed after UV processing.

**Statistical analysis**

The heatMap package (Version 1.0.12R), Graphpad Prism (Version 8.0.1), and Cytoscape (Version 3.8.0) software were used for graphical and statistical analysis. Statistical significance was determined using the student T-test (two-sided), with error lines in the graph data representing the mean standard deviation. The threshold for differential screening was with the absolute value of FC > 4 and *p* < 0.01. The correlation coefficient threshold was 0.8. The minimum interaction score was set at 0.9 (high confidence), and Cytocubba (Cytoscape plug-in) identified the most relevant nodes in the network by degree.

**Acknowledgments**

The work was primarily supported by a research fund provided by the Zhejiang Provincial Natural Science Foundation (LY22H120002), the Zhejiang Provincial and Ministry of Health Research Fund for Medical Sciences (WKJ-ZJ-1910), and the Wenzhou Basic Research Projects (Y2020916).

**Ethics approval and consent to participate**

The procedures used in this study for the collection and treatment of clinical samples of pancreatitis were approved by the Scientific Research Ethics Review Committee of the First Affiliated Hospital of Wenzhou Medical University.

**Authors' contributions**

QZ and RY contributed equally to this work. FL designed the study. RY, KS, and HL designed and performed experiments with the assistance of JL, HX, HL, and XL. KS and HL provided critical materials and reagents for the experiments. QZ. And RY contributed to the writing of the manuscript and interpretation of data. FL supervised all the experiments in this study. All authors read and approved the final manuscript.


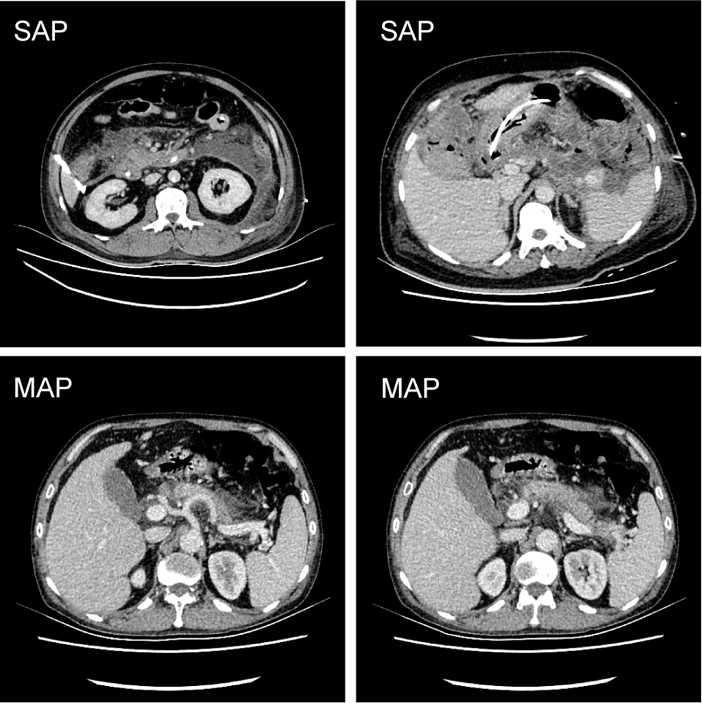


**Figure S1**. Comparison of CT images of MAP and SAP patients.


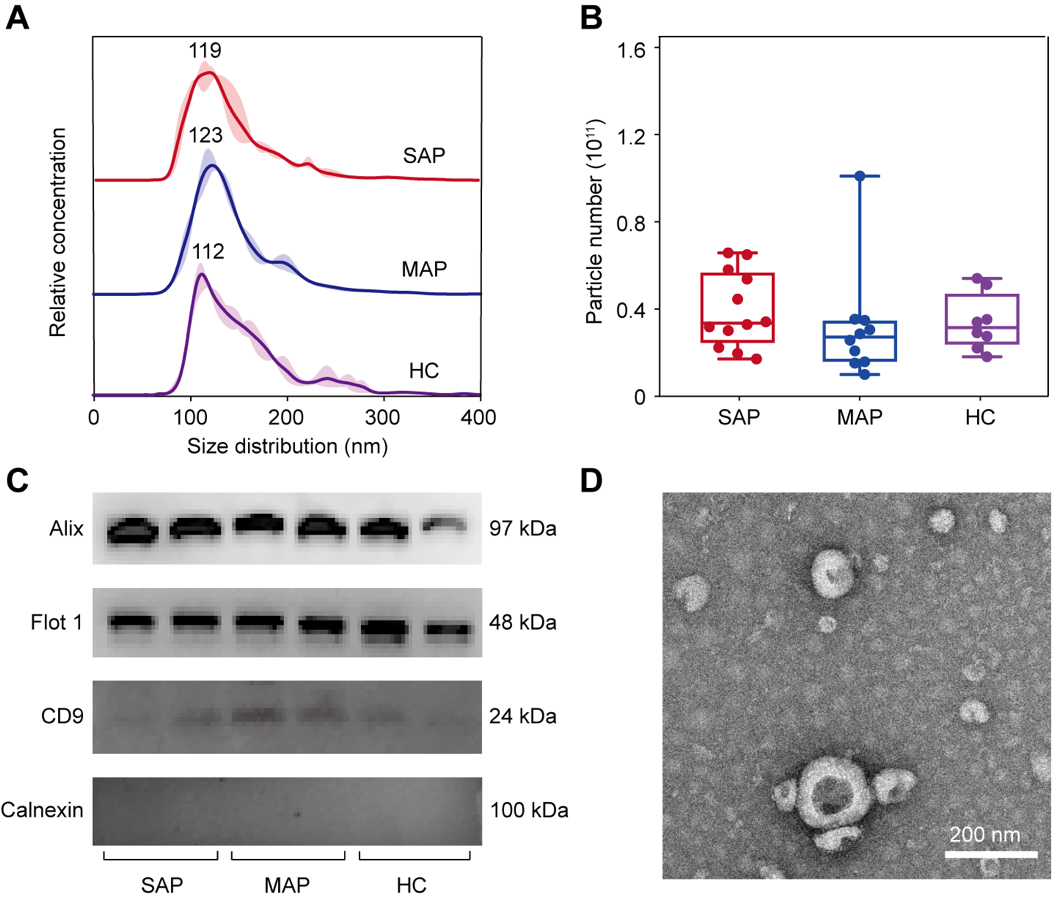


**Figure S2.** Characterization of plasma-derived sEV. **(A)** NTA analysis of sEV from severe acute pancreatitis, mild acute pancreatitis, and healthy controls. **(B)** The number of isolated particles from 200 μL of plasma samples. **(C)** Western Blot analysis of EVs protein markers in severe acute pancreatitis, mild acute pancreatitis, and healthy controls. **(D)** Typical EV morphology by transmission electron microscopy.


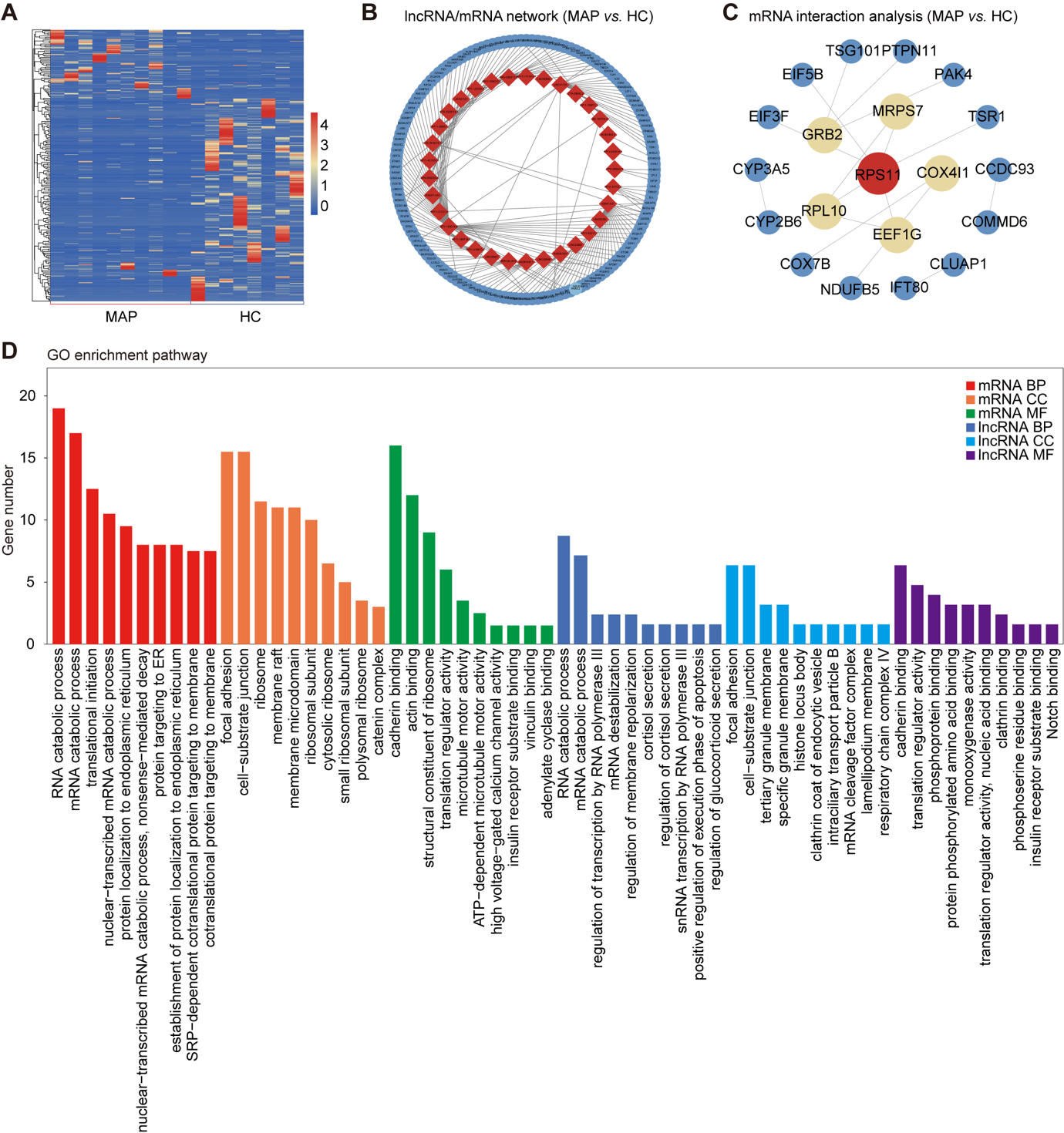


**Figure S3.** Differential analysis of gene expression profiles between MAP and HC. (**A)** Cluster heat map showing the differential genes between MAP and HC. **(B)** LncRNA and mRNA interaction network based on the differential genes between MAP and HC. Diamond and red: lncRNA; Round and blue: mRNA. The correlation coefficient threshold was 0.8. **(C)** Interaction network diagram of target genes of lncRNA. The circles from large to small represent the order of importance in the topology. **(D)** The GO enrichment pathway analysis is based on the differential genes between MAP and the HC.

**
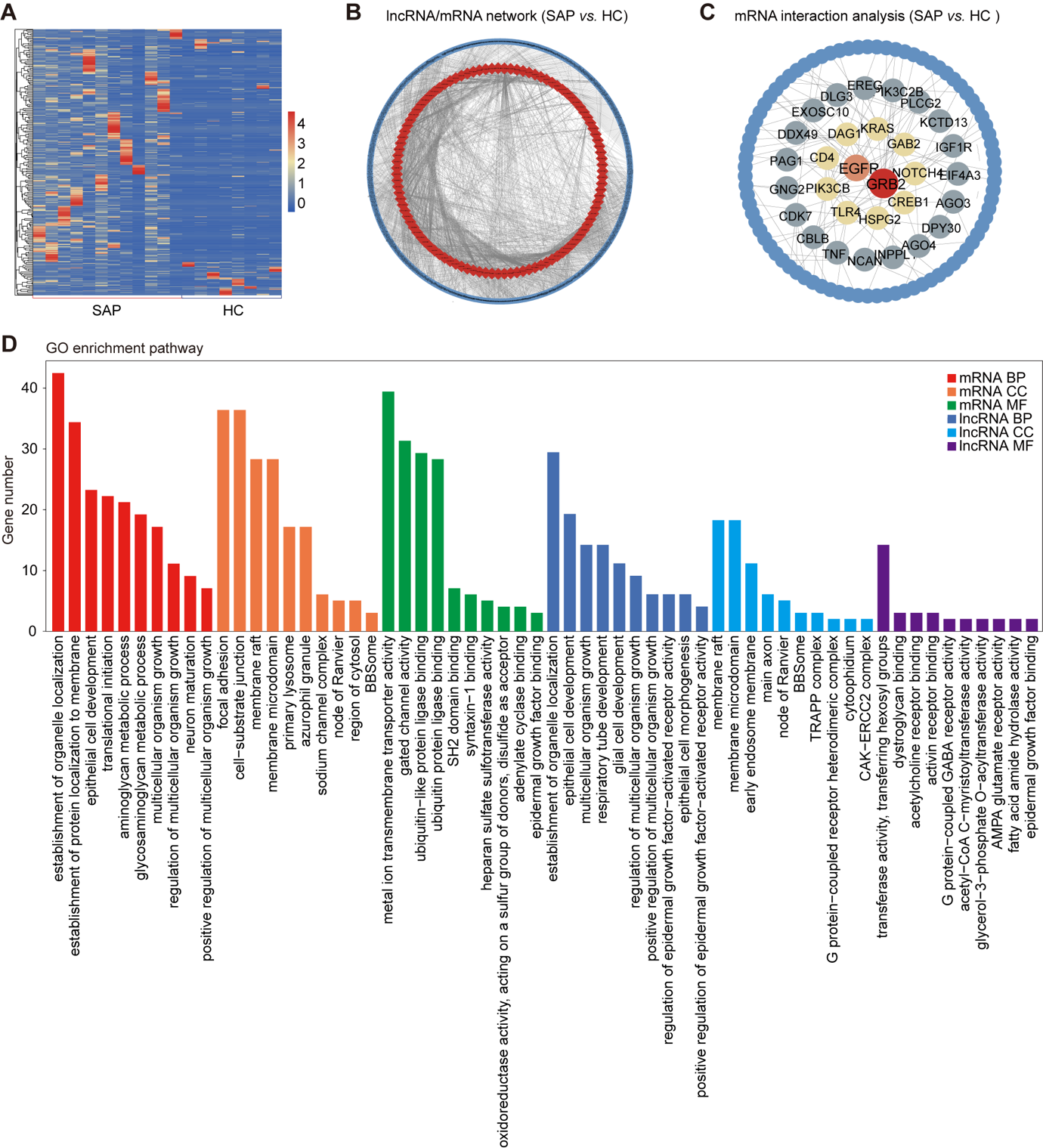
**

**Figure S4.** Differential analysis of gene expression profiles between SAP and HC. **(A)** Cluster heat map showing the differential genes between SAP and HC. **(B)** LncRNA and mRNA interaction network based on the differential genes between SAP and HC. Diamond and red: lncRNA; Round and blue: mRNA. The correlation coefficient threshold was 0.8. **(C)** Interaction network diagram of target genes of lncRNA. The circles from large to small represent the order of importance in the topology. **(D)** The GO enrichment pathway analysis based on the differential genes between SAP and HC.


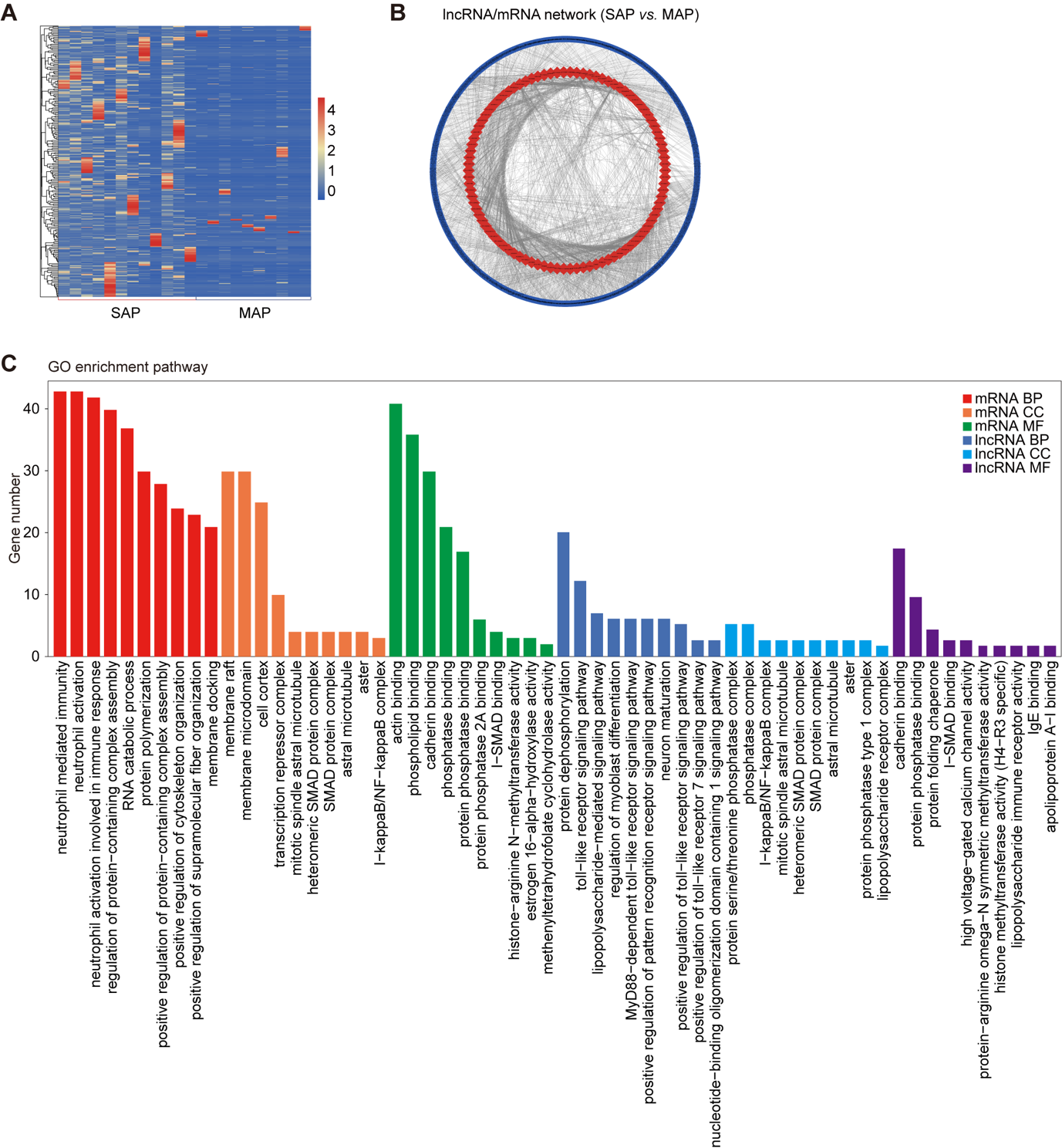


**Figure S5.** Differential analysis of gene expression profiles between MAP and SAP. **(A)** Cluster heat map showing the differential genes between SAP and MAP. **(B)** LncRNA and mRNA interaction network based on the differential genes between SAP and MAP. Diamond and red: lncRNA; Round and blue: mRNA. **(C)** The GO enrichment pathway analysis based on the differential genes between the SAP and MAP.

**Table S1.** Clinical statistics of acute pancreatitis patients**.**

|  | SAP  (n=12) | MAP  (n=10) | Healthy control  （n=8） |
| --- | --- | --- | --- |
| Total | 40% | 33.3% | 26.6% |
| Median  (range) | 32.5  (21-46) | 38.5  (26-50) |  |
| Men | 10 | 5 |  |
| Women | 2 | 5 |  |

SAP: severe acute pancreatitis; MAP: mild acute pancreatitis;

**Table S2.** Top 20 differential mRNA and top 20 differential lncRNA between MAP and HC group (*p* value ascending order).

| **mRNA** | ***P* value** | **Fold change** | **lncRNA** | ***P* value** | **Fold change** |
| --- | --- | --- | --- | --- | --- |
| TUBA1B | 3.18E-23 | -11.81 | CTD-2555A7.2 | 2.82E-08 | -6.06 |
| EEF1G | 1.47E-15 | -11.46 | RP11-247C2.2 | 3.08E-08 | -6.69 |
| RPL10 | 1.10E-18 | -10.51 | GDNF-AS1 | 4.02E-08 | -6.11 |
| BTF3 | 3.23E-18 | -11.80 | RP11-326I11.3 | 9.68E-08 | 6.63 |
| COMMD6 | 1.21E-16 | -9.30 | LINC00282 | 1.03E-07 | -5.99 |
| ARHGDIB | 2.97E-16 | -11.23 | RP11-95D17.1 | 3.46E-07 | -6.70 |
| HNRNPA1 | 6.33E-16 | -11.05 | LINC00687 | 4.66E-07 | -5.45 |
| TUBA1A | 9.06E-16 | -9.23 | RAB30-AS1 | 2.03E-06 | -4.91 |
| GIMAP4 | 1.34E-15 | -10.71 | LINC00959 | 3.14E-06 | -5.99 |
| KMT2E | 3.10E-14 | -9.77 | AC087430.1 | 3.85E-06 | -4.95 |
| LST1 | 4.53E-14 | -9.702 | MIR194-2HG | 5.19E-06 | -4.98 |
| CRK | 1.49E-13 | -9.98 | RP11-596C23.2 | 9.58E-06 | 5.30 |
| ZYX | 5.44E-13 | -9.53 | LINC00567 | 1.15E-05 | -4.29 |
| RNF7 | 1.76E-12 | -9.16 | LINC01427 | 1.47E-05 | -5.20 |
| B4GALNT1 | 3.22E-12 | -8.84 | RP11-318K12.2 | 2.04E-05 | -4.45 |
| MYD88 | 3.88E-12 | -8.70 | RP11-185P18.2 | 3.75E-05 | -4.33 |
| GPX3 | 9.54E-12 | -8.33 | RP11-56D16.8 | 4.47E-05 | -4.35 |
| CDON | 1.00E-11 | 9.02 | RP11-212I21.4 | 4.75E-05 | -5.10 |
| TCP1 | 1.05E-11 | -9.06 | PAXIP1-AS1 | 5.32E-05 | 5.05 |
| MSMO1 | 1.35E-11 | -8.55 | RP4-555D20.4 | 5.90E-05 | -4.09 |

**Table S3.** Top 20 differential mRNA and top 20 differential lncRNA between SAP and HC group (P value ascending order).

| **mRNA** | ***P* value** | **Fold change** | **lncRNA** | ***P* value** | **Fold change** |
| --- | --- | --- | --- | --- | --- |
| C19orf53 | 3.28E-15 | -9.56 | RP11-95D17.1 | 8.59E-10 | -7.28 |
| NRXN1 | 1.43E-11 | 7.74 | LINC01568 | 9.07E-10 | 7.69 |
| TPO | 2.41E-11 | 6.80 | RP11-95O2.1 | 1.72E-09 | 6.61 |
| GCA | 3.88E-11 | 8.35 | LINC01102 | 2.17E-09 | 6.39 |
| TAF1C | 3.99E-11 | 7.95 | RP11-214K3.24 | 3.65E-09 | 5.97 |
| DRG2 | 4.91E-11 | 8.51 | RP11-755B10.3 | 3.97E-09 | 6.69 |
| TMEM232 | 6.67E-11 | 7.08 | BSN-AS2 | 1.97E-08 | 6.67 |
| ESAM | 6.88E-11 | 8.68 | RP11-380J14.1 | 4.16E-08 | 5.65 |
| RPL10A | 8.84E-11 | -6.07 | LINC01435 | 7.44E-08 | 6.52 |
| LRP8 | 1.24E-10 | 7.36 | RP11-849I19.1 | 7.66E-08 | 5.98 |
| QRICH1 | 1.30E-10 | 7.83 | BISPR | 1.36E-07 | 6.36 |
| TMEM236 | 2.54E-10 | 7.58 | LINC00606 | 1.43E-07 | 6.39 |
| ZNF557 | 2.57E-10 | 6.11 | RP5-984P4.6 | 1.62E-07 | 6.11 |
| ILKAP | 3.38E-10 | 7.05 | RP11-890B15.2 | 2.28E-07 | 5.21 |
| BST2 | 4.26E-10 | -8.09 | RP11-352M15.2 | 2.91E-07 | 4.92 |
| NSMCE4A | 4.44E-10 | 7.70 | RP13-631K18.3 | 3.36E-07 | 5.72 |
| BCL7A | 4.55E-10 | 7.01 | RP11-88E10.4 | 3.39E-07 | 5.02 |
| OLFML2A | 4.56E-10 | 7.10 | RP11-3P17.5 | 3.76E-07 | -4.11 |
| COMMD9 | 4.99E-10 | 7.43 | RP11-3B7.1 | 4.38E-07 | 6.06 |
| MYO1F | 5.26E-10 | 7.07 | PWRN1 | 4.90E-07 | 4.99 |

**Table S4.** Top 20 differential mRNA and top 20 differential lncRNA between SAP and MAP group (*p* value ascending order).

| **mRNA** | ***P* value** | **Fold change** | **lncRNA** | ***P* value** | **Fold change** |
| --- | --- | --- | --- | --- | --- |
| TUBA1B | 2.25E-19 | 9.28 | LINC00152 | 7.40E-17 | 8.44 |
| S100A11 | 3.04E-17 | 8.97 | LINC01304 | 2.13E-11 | 7.65 |
| ARHGDIB | 4.32E-17 | 10.34 | AC104024.1 | 4.92E-11 | 7.41 |
| MIF | 5.73E-17 | 9.41 | RP11-147L13.11 | 2.65E-10 | 6.69 |
| OAS3 | 5.47E-14 | 8.25 | RP11-71N10.1 | 2.90E-10 | 6.02 |
| SERPINB1 | 7.47E-14 | 8.58 | RP11-134D3.1 | 3.23E-10 | 6.87 |
| RPL10 | 2.91E-13 | 7.55 | SRP14-AS1 | 3.32E-10 | 7.37 |
| SLC25A42 | 4.35E-13 | 7.76 | LINC00958 | 8.98E-10 | 6.11 |
| C16orf54 | 1.02E-12 | 6.14 | RP11-849I19.1 | 3.81E-09 | 5.97 |
| UBL5 | 1.06E-12 | 6.96 | RP11-88E10.4 | 7.53E-09 | 5.25 |
| KMT2E | 1.52E-12 | 7.65 | RP11-14C10.6 | 9.18E-09 | 6.04 |
| TTPAL | 2.00E-12 | 6.31 | RP11-795F19.1 | 1.47E-08 | 5.68 |
| ZFYVE27 | 2.06E-12 | 7.33 | RP11-327I22.5 | 1.72E-08 | 5.55 |
| TIMM23 | 2.50E-12 | 7.08 | KCNIP4-IT1 | 2.15E-08 | 5.82 |
| ZYX | 3.32E-12 | 7.81 | SPACA6P-AS | 2.62E-08 | 5.81 |
| GYG1 | 4.10E-12 | 8.03 | LINC00964 | 7.72E-08 | 5.79 |
| UBC | 5.10E-12 | 8.04 | CTB-25B13.5 | 1.16E-07 | 4.98 |
| MRPL16 | 5.40E-12 | 6.97 | RP11-570K4.1 | 1.33E-07 | 5.15 |
| DESI1 | 6.18E-12 | 6.33 | AC069513.4 | 1.64E-07 | 5.28 |
| NEK7 | 1.00E-11 | 7.62 | FAM95C | 1.84E-07 | 5.42 |

**Table S5.** Differential metabolites between SAP and MAP (*p* < 0.05; FC > 1.2)

| **Name** | **Class** | ***P* value** | **Fold change** |
| --- | --- | --- | --- |
| 2-(Methylthio)benzothiazole | Benzene and substituted derivatives | 1.75524E-06 | 2.07 |
| 2,6-Di-tert-butyl-4-methylphenol | Benzene and substituted derivatives | 0.035507361 | 1.55 |
| Palmitaldehyde | Lipids Fatty Acids | 3.89067E-05 | 1.52 |
| Hyodeoxycholic Acid | Bile Acids | 0.000928718 | 1.465 |
| 2,5-Dimethyl pyrazine | Heterocyclic compound | 0.003727066 | 1.43 |
| 1-Naphthol | Phenols And Its Derivatives | 0.000727504 | 1.42 |
| 1-Octen-3-one | Ketones | 0.006651306 | 1.38 |
| 2,6-Dimethylpyridine | Pyridine And Pyridine Derivatives | 0.01116041 | 1.38 |
| 6-Phosphogluconic Acid Trisodium Salt | Carbohydrate metabolomics | 0.007006068 | 1.34 |
| Cholesterol | Lipids | 0.001933978 | 1.33 |
| p-Menth-1-en-4-ol | Terpenoid | 0.011049169 | 1.32 |
| 9-Fluorenone | Ketones | 0.014379505 | 1.31 |
| Cis-11,14,17-Eicosatrienoic Acid (C20:3) | Lipids Fatty Acids | 0.000305516 | 1.28 |
| (+)-borneol | Alcohol | 0.015117815 | 1.28 |
| Dopamine | Polyamine | 0.032802535 | 1.27 |
| 6-Methyl-5-hepten-2-one | Ketones | 0.01840627 | 1.27 |
| Diphenyl ether | Benzene and substituted derivatives | 0.032816038 | 1.25 |
| Hydrocinnamic Acid | Organic Acid And Its Derivatives | 0.010415184 | 1.25 |
| Triethyl phosphate | Organic Acid And Its Derivatives | 0.02252712 | 1.24 |
| 4-Ethylbenzoic Acid | Benzene and substituted derivatives | 0.00148601 | 1.24 |
| alpha-Terpinene | Terpenoid | 0.00206884 | 1.22 |
